# Antiviral Properties of Polyphenols from Plants

**DOI:** 10.3390/foods10102277

**Published:** 2021-09-26

**Authors:** Katarzyna Chojnacka, Dawid Skrzypczak, Grzegorz Izydorczyk, Katarzyna Mikula, Daniel Szopa, Anna Witek-Krowiak

**Affiliations:** Department of Advanced Material Technologies, Faculty of Chemistry, Wrocław University of Science and Technology, Smoluchowskiego 25, 50-372 Wrocław, Poland; katarzyna.chojnacka@pwr.edu.pl (K.C.); dawid.skrzypczak@pwr.edu.pl (D.S.); katarzyna.mikula@pwr.edu.pl (K.M.); daniel.szopa@pwr.edu.pl (D.S.); anna.witek@pwr.edu.pl (A.W.-K.)

**Keywords:** polyphenol, antiviral properties, polyphenols isolation, polyphenols determination

## Abstract

Polyphenols are active substances against various types of viral infections. Researchers have characterized methods of how to isolate polyphenols without losing their potential to formulate pharmaceutical products. Researchers have also described mechanisms against common viral infections (i.e., influenza, herpes, hepatitis, rotavirus, coronavirus). Particular compounds have been discussed together with the plants in the biomass in which they occur. Quercetin, gallic acid and epigallocatechin are exemplary compounds that inhibit the growth cycle of viruses. Special attention has been paid to identify plants and polyphenols that can be efficient against coronavirus infections. It has been proven that polyphenols present in the diet and in pharmaceuticals protect us from viral infections and, in case of infection, support the healing process by various mechanisms, i.e., they block the entry into the host cells, inhibit the multiplication of the virus, seal blood vessels and protect against superinfection.

## 1. Introduction

Since time immemorial, plants have been sources of valuable bioactive substances used worldwide to treat various diseases. Isolation and characterization of compounds present in herbs has contributed to the discovery of new sources of pharmaceuticals. About 11% of essential drugs available globally come exclusively from plants [1]. Contrary to their synthetics, natural preparations are anti-inflammatory, antioxidant, and anticancer in different ways and they are safer for patients because they have no side effects [2].

Polyphenols—secondary plant metabolites that constitute a group of over 8000 structural variants—are of great interest in this respect. Depending on the number of rings and elements connecting these rings, they are classified as flavonoids, phenolic acids, stilbenes or lignans [3]. Pérez-Jiménez et al. (2010) identified the top 100 dietary sources of polyphenols: spices, fruits, seeds, vegetables, drinks and oils. Most of these bioactive compounds are found in cloves (150 mg/g) and peppermint (112 mg/g). The polyphenol content in fruits (red raspberry, blackberry, strawberry, apple) ranges from 1.5 to 3 mg/g of fresh weight. Vegetables rich in bioactive compounds include black or green olives (5.69 mg/g and 3.46 mg/g, respectively), artichokes (2.6 mg/g) and chicory (2.35 mg/g). Tea (black 102 mg/100 mL, green 89 mg/100 mL), red wine (101 mg/100 mL) and rapeseed oil (92 mg/100 mL) can also be sources of polyphenols [4]. Some of the many factors that determine the content of bioactive compounds are light exposure, soil and climatic conditions, type of cultivation (greenhouses, fields, biological or hydroponics), maturity during harvest, storage and processing (e.g., peeling, cooking) [5].

In vitro and in vivo tests show that polyphenols support anti-inflammatory functions and the human body’s defense by modulating immune regulation and inhibiting cytokine storms; they also inhibit proinflammatory cytokines, model cellular immunity and act as immunomodulators. They act as free radical scavengers in which they are aided by micronutrients and vitamins. All the desirable effects depend on their bioavailability and the amount consumed [6]. 

New and mutating viruses require new pharmaceuticals. Especially, during the ongoing COVID-19 pandemic and due to the lack of efficient treatment, scientists are trying to develop new preparations to inhibit the penetration and replication of pathogens from the coronavirus family, especially the spread of SARS-CoV-2 (severe acute respiratory syndrome) [7]. Having collected data from scientific sources, patents and clinical trials, Song et al. (2021) have shown that Chinese herbal medicines (mixtures containing, among others, ginger, ginkgo, sage) can block the enzymatic activity of 3CLpro [8]. Acorns and their extracts can also inhibit SARS-CoV-2 replication due to high concentrations of gallic acid [9]. Indian herbs commonly used as food or nutraceuticals (turmeric, nettle, garlic, sage, aloe and black tea) also show activity against virus RNA [10]. 

Below, we report up-to-date literature on the potential antiviral activity of polyphenols isolated from plants (terrestrial and aquatic), emphasizing the mechanisms and biological basis of these compounds. We know of no publications detailing the mechanisms of action of polyphenols and their impact on the course of viral infections, not only coronaviruses but also influenza, herpes, Epstein–Barr hepatitis, rotavirus and adenovirus. Later in the text, we discuss the latest methods enabling the effective isolation of polyphenols from plants and their identification techniques.

## 2. Polyphenol Effectiveness against Viral Infections

### 2.1. Influenza Viruses

Influenza is caused by RNA *Orthomyxoviridae* viruses containing an envelope with two glycoproteins (hemagglutinins (HA) and neuraminidase (NA)) and an ion channel (M2). Viruses are divided into A, B, C and D types. Type A is divided into subtypes based on the nature of two proteins on the virus surface: HA and NA [11]. The influenza virus predominantly attacks the respiratory system, causing its failure and numerous complications in the form of bacterial infections. Periodically reoccurring influenza epidemics are responsible for a large percentage of deaths [12]. Frequent mutations of the virus make vaccines ineffective. The emergence of strains that are resistant to antiviral drugs, ion channel inhibitors (e.g., amantadine) and NA inhibitors (e.g., oseltamivir) compels us to look for alternative compounds. Influenza leads to oxidative stress, which—if uninhibited by antioxidants—produces large amounts of free radicals, damaging lung tissue [13]. Phytotherapy exploits the natural antioxidants which are food components of fruits and vegetables (vitamin C, polyphenols, carotenoids) [14]. Polyphecatechnols, which are primary components of fruits and beverages (green tea, wine), can be used to prevent and treat influenza, as demonstrated in many in vitro and in vivo studies. 

Berries are a rich source of various bioactive compounds with antiviral activity [15]. Black elderberry, *Sambucus nigra*, is an ingredient of Sambucol preparation with proven anti-influenza efficacy [16]. Blackcurrant extracts, apart from inhibiting adsorption and replication of the virus, reduce the formation of complications, including inhibition of other pathogenic microorganisms [17]. Blueberries and cranberries have strong antiviral effects due to the presence of polyphenols [18].

*Aronia melanocarpa* ethanol extract contains antiviral polyphenols (like ellagic acid) and flavonoids (like myricetin, kaempferol and quercetin). Compounds isolated from chokeberry showed high anti-influenza efficacy: even an amount of 0.0625 mg inhibited almost 70% of seasonal influenza viruses of H1 and H3 type. Aronia extract is most likely to inhibit virus surface proteins (mainly HA) [12].

Polyphenols extracted from the brown alga *Ecklonia cava* are rich in phlorotannins (including phloroglucinol, ecol, 7-phloracol, fluorofucofuroecol and diecol) and appear to be a selective NA inhibitor of the influenza virus. The ethanolic extract showed very strong NA inhibitory activity (72.1% inhibition at 30 μg/mL). The IC_50_ for phlorofucofuroeckol was 4.5 µM, which was the highest of all components. NA is an important target for antiviral agents. The inhibition of this enzyme causes viruses to aggregate, which stops the progression of infection [19].

The ethanolic extract of the aerial roots of *Geranium sanguineum* has been used in folk medicine to treat various infections. It contains a high amount of phenolic compounds (35% soluble), especially tannins, flavonoids, catechins and proanthocyanidins type A and B (A-PAC, B-PAC). Studies on mice have shown that these compounds significantly affect liver metabolism, reducing the effects of oxidative stress during influenza virus infection [13].

The polyphenols present in the aerial parts of *Eupatorium perfoliatum*, isolated in water–alcohol extract, effectively treat influenza. Their activity is associated with blocking the attachment of the virus to the cell surface [20].

The juice and seeds of *Morus alba* contain a number of polyphenols. Cyanidin-3-rutinoside (nearly 30 mg/g), cyanidin-3-glucoside, rutin and gallic acid have been identified in the juice. Aqueous and alcoholic extracts exhibit inhibitory activity against the H1N1 influenza virus in a dose-dependent manner. Presumably, the active compounds inhibit viral attachment to cells or affect the inhibition of viral entry into cells [11]. 

Pomegranate fruit is a traditional remedy with a range of health benefits. The main active compounds are polyphenols: tannins and flavonoids. It is hypothesized that polyphenols bind to virus surface glycoproteins, which changes the molecular structure of these proteins and thus antigenic determinants [21].

### 2.2. Hepatitis Viruses

Five types of viruses cause hepatitis, its attendant complications and chronic liver disease, most commonly hepatitis A virus (HAV), hepatitis B virus (HBV) and hepatitis C virus (HCV). Depending on the type, the viruses are transmitted by food, sex organs and blood, and may also cause co-infections. Vaccines against these viruses are not widespread. Treatment consists of administering immunomodulators (interferon) or nucleoside and nucleotide analogs, which can cause several side effects or result in drug resistance [22]. New antiviral strategies based on natural compounds are being sought. Non-cytotoxic compounds at doses of 2.5–50 µg/mL have been tested in vitro as novel HBV inhibitors. Maximum inhibition of HBsAg antigen on day 5 was obtained for quercitin (73%) [23]. Studies show that quercetin inhibits genome replication in human cells, and coincubation with nucleo-side analogs (lamivudine, entecavir, or adefovir) enhances quercetin’s efficacy against HBV [24].

Green tea polyphenols, particularly epigallocatechin and epigallocatechin-3-gallate (EGCG), epicatechin and epicatechin gallate (ECG), have antiviral activity. Specifically, EGCG, which represents half of the green tea polyphenol fraction, has broad inhibitory activity against various viruses, including hepatitis. This compound has been shown to inhibit HCV’s entry into cells via viral envelope proteins and inhibit cell-to-cell transmission [25]. The IC_50_ inhibitory concentration is 5 µm and the IC_90_ inhibitory concentration is approximately 50 µm for recombinant HCV (JFH1-Luc). The compound can be coupled with other antiviral drugs [26]. Theaflavins in black tea also have an inhibitory effect on HCV by acting directly on the virus before it enters cells [27]. Tannic acid, present in many plants, has a similar effect and has been shown to inhibit Huh7.5 cell entry at an IC_50_ concentration of 5.8 µM [28].

The main mechanism of antiviral action of EGCG is inhibition of DNA synthesis during HBV replication [29]. This compound’s effect, which is strongly dose-dependent, was observed at concentrations of 0.11–0.44 µmol/mL for HepG2 2.2.15 cells compared to the drug lamivudine (0.87 µmol/mL) [30]. Most studies on the antiviral activity of polyphenols involve in vitro or cell line studies. Clinical trials are needed to evaluate the efficacy of these compounds and to select a therapeutic dose.

The antioxidant resveratrol is responsible for activating the longevity gene (sirtuin). Due to it, consumption of red wine is considered beneficial. It is known that resveratrol is recommended as an adjunctive supplement to the treatment of chronic HCV. However, cellular studies have examined the antioxidant’s effect on the antiviral effects of interferon and have reported that resveratrol is not recommended as a supplement in antiviral therapy because it significantly enhances virus replication [31]. Similarly, in the case of HBV virus, resveratrol strongly affects the core promoter and enhances transcription and replication of the virus [32]. Studies have also demonstrated that resveratrol multimers strongly inhibit HCV replication. Resveratrol dimers ((+)-ε-viniferin) extracted from grapevine root and chemically synthesized (with or without acetylation) were found to be a promising group of novel anti-HCV drugs with minimal cytotoxicity [33].

Curcumin as a natural compound has antiviral properties and it can be used to treat HBV patients by targeting cellular and metabolic pathways (adhesion molecule, protein kinases, inflammatory cytokine, transcription factors, anti-apoptotic proteins). Curcumin can activate PPAR-γ receptor in adipose tissue, increase cellular glutathione content and modify the level of PGC-1α protein that regulates gluconeogenesis in the liver, leading to downregulation of transcription factor NF-κB [34]. 

Cellular studies have confirmed the effectiveness of curcumin in inhibiting HBV expression. A dose of 20 µmol/L curcumin applied for 2 days reduced HBsAg antigen levels to 57.7% [35]. Animal studies (rats, guinea pigs, monkeys) have shown that curcumin is safe in most cases. In clinical studies (25 subjects), 8000 mg of curcumin was dosed daily and no toxic effects of the substance were observed [34].

### 2.3. Herpes Virus

The herpes virus belongs to the *Herpesviridae* family, which also includes Epstein–Barr, cytomegalovirus and varicella zoster viruses. This virus has a double-stranded DNA and is an enveloped virus that, once inside, cannot be removed from the body and enters the cells of the nervous system, where it can survive asymptomatically for many years. When exposed to unfavorable conditions (stress, immunodeficiency), it becomes active and again manifests disease symptoms. The most common are herpes simplex virus 1 (HSV-1), which causes infections around the mouth, and herpes simplex virus 2 (HSV-2), responsible for infections of reproductive organs [36]. The most common drugs against this virus are nucleoside analogs, such as acyclovir, which inhibits DNA polymerase.

Active components of hibiscus extracts show potent activity against the HSV-1 virus, indicating the blockade of virus replication and prevention of virus–cell interaction in vitro [37]. Similarly, the tea polyphenol EGCG inhibits the cytotoxic effect of the virus on cell viability and viral protein expression in amounts as low as 25 µg/mL. This may be due to the effects of polyphenols on virions before they enter the cell [38]. Polyphenolic extracts of pistachio kernels showed synergistic effects of all the components with antiviral properties. The antiviral activity of EC_50_ against HSV-1 was in the amount of 0.4 mg/mL in plaque assay [39]. The strong anti-herpes activity was proven for plant extracts from *Euphorbia coopire* (EC_50_ 36 ng/mL) and *Morus alba* (EC_50_ > 50 µg/mL), in which several flavonoid compounds were found, including quercetin [40]. 

Silver nanoparticles stabilized with tannic acid may be an effective agent against genital herpes. The antiviral nature of both plant extracts and nanoparticles is exploited here, indicating a multidirectional mechanism of action associated with blocking virus attachment and producing compounds with anti-HSV-2 activity (cytokines, chemokines) [41].

### 2.4. Epstein–Barr Virus

A high percentage of the population carries Epstein–Barr virus but shows no symptoms of the infection throughout their lives. Infectious mononucleosis, caused by this virus, mainly affects teenagers and young adults, mostly immunocompromised individuals [42]. In AIDS patients, Epstein–Barr virus can cause hairy leucoplakia of the tongue. The virus has also been associated with malignancies such as Burkitt’s lymphoma, Hodgkin’s lymphoma and nasopharyngeal carcinoma [43]. The main targets of the virus are resting B lymphocytes, which are induced to proliferate by the virus. The viral cycle allows the horizontal spread of the virus and promotes the development of B-cell nodules [44]. Treatment of individuals with symptoms of virus infection mainly involves the use of pharmacotherapy and, in more complex cases, chemotherapy or radiation. The use of antiviral compounds is associated with a high likelihood of side effects. In most cases, such drugs have poor bioavailability, which limits their impact [45]. 

The literature has shown that some polyphenols have properties that inhibit the expression of Epstein–Barr Virus lytic genes [46]. An investigation was conducted into the antiviral activity of resveratrol, a polyphenol contained in many plant species and their fruits (blackcurrant, strawberries, raspberries, mulberries or grapes). Resveratrol is a bioflavonoid compound that exhibits antibacterial, antifungal, anticancer and antioxidant properties [47]. Resveratrol significantly inhibits the expression of virus lytic genes. The inhibition of its reproduction is strictly dependent on the dose of polyphenol. Resveratrol decreases levels of reactive oxygen species, blocks protein synthesis and inhibits virus-induced activation of transcription factors, which affects Epstein–Barr virus replication in individuals [42]. Among a large group of polyphenols, high effectiveness against Epstein–Barr virus is reported for luteolin, which is used to treat hypertension and inflammatory disorders in Chinese medicine. The flavonoid was tested for its ability to inhibit virus reactivation. Luteolin stops the expression of proteins from lytic genes and B EBV-positive cells. Additionally, the study showed reduced viral production and a reduced number of virus reactivating cells [48].

Literature data present a method of synthesis of new polyphenols based on esters consisting of naturally occurring materials: ferulic acid (mainly from rice and wheat) and gallic acid (from a commercial basis) [49]. Studies have been carried out on the inhibitory effect of polyphenol on 12-tetradecanoylphorbol-13-acetate, which activates the Epstein–Barr virus. The authors successfully demonstrated the effectiveness of the new polyphenols. The production of ferulic acid (from rice bran) and gallic acid is already commercialized, theoretically allowing for broader manufacturing and use of high-activity antiviral preparation. Components of natural origin are also being used as building blocks to produce biocompatible forms to treat the Epstein–Barr virus. A nanovaccine has been prepared from tannic acid and a novel protein antigen to fight virus-associated cancer. The polyphenol, as a plant-derived material, self-organizes with antigens and adjuvants to form an effective nanoparticle vaccine. Nanovaccine, targeting lymph nodes, together with CpG adjuvant, causes immune activation. This induces effective inhibition of oncogenesis. In studies on mice, it has been shown that the application of the agent also results in a decrease in the tumor and an increase in the lifespan of the rodents [50].

### 2.5. Rotavirus

Rotavirus is a non-motile virus that contains a multilayered virion that consists of 11 RNA segments. This virus is a major pathogen that causes gastroenteritis and acute diarrhea, which leads to dehydration. As many as 200,000 deaths per year are caused by rotavirus infection. Available information indicates no clear evidence of the presence of an effective treatment for the infected individual. Recovery is mainly based on replenishing fluids and electrolytes. The duration of the infection can be shortened by probiotics [51]. The global rotavirus problem can be reduced with the two available vaccines (Rotarix and RotaTeq), but the live form of the vaccines, the high risk of transmission and variations in efficacy limit their application [52].

Polyphenols isolated from the roots of *Glycyrrhiza uralensis* plants inhibit rotavirus from binding with cells and replicating. This effect of extracted polyphenols (licocoumarone, 2′-methoxyisoliquiritigenin, glyasperin C, glycyrin, licoflavono and glyasperin D) on rotavirus of the G5P and G8P group has been proven in vitro tests. It has been shown that resveratrol at a dose of 20 µM inhibits rotavirus replication in the Caco-2 cell line [52]. The bioflavonoid inhibits RNA synthesis, protein expression or the formation of viroplasmic plaques. Research on mice treated with resveratrol showed that the agent reduced diarrhea’s severity and alleviated other symptoms [53]. High antiviral activity was also confirmed for *Myracrodruon urundeuva* leaves. Plant extracts were tested for inhibition of monkey rotavirus SA-11, showing 75–92% virucidal effect [54]. 

*Achyrocline bogotensis*, grown in Colombia, is widely used for the treatment of skin, respiratory and urinary tract infections. The high anticancer activity of flavonoids obtained from this plant has also been proven in some papers. A C6-C3-C6 phenylbenzopyrone backbone characterizes a large proportion of these compounds. The efficacy of extracts derived from *Achyroklina bogotensis* was tested in treating viral gastrointestinal diseases caused by rotavirus and astrovirus [55]. The study confirmed high antiviral and antimicrobial efficacy. Anti-inflammatory and antiviral effects were also demonstrated by *Sophora flavescens* root extract containing the flavone norkurarinol. Norkurarinol suppresses the mRNA expression of proinflammatory and adhesion molecules caused by poly(I:C) and rotavirus infection. In addition, the flavonoid inhibits cytoplastic effects induced by rotainfection. Norkurarinol modulates inflammatory responses mediated by toll-like receptor 3 and rotavirus proliferation [56].

### 2.6. Coronavirus 

The potential of polyphenols was underlined during the SARS-CoV-2 pandemic because they present multiple defense mechanisms against coronaviruses. This is a group of RNA viruses transmitted mainly by birds and mammals. The name derives from the image of the virus obtained under the electron microscope, in which the “crowns” of the virus envelope are visible. The best-known viruses are SARS-CoV-1 and SARS-CoV-2, responsible for severe acute respiratory syndrome (SARS) [57].

The effect of polyphenols on coronavirus infections is complex. First of all, selected polyphenols—e.g., luteolin—show a high affinity with the S protein of the virus, thus preventing its entry into human cells [58]. Inhibition of the S protein is mainly caused by polyphenols present in citrus fruits, turmeric and rhubarb roots. Other scientific sources mention the possibility of blocking protein S by active compounds contained in herbs and tea-naringenin, EGCG or herbacetin [59]. Entry into host cells is also blocked by blocking the enzyme ACE2, which is the entry point of SARS-CoV-2. Polyphenols in turmeric, yerba and red grapes (eriodicytol, resveratrol, curcumin and catechin), which have a high affinity for ACE2 ligands, are beneficial for this purpose [60].

After the virus enters the human body with the help of polyphenols, its multiplication by RNA replication is blocked. Actions are mainly directed at protease inhibition, thus blocking transcription and replication of genetic material. Here also the role of polyphenols from turmeric and citrus fruits has been appreciated [60]. On the other hand, the polyphenols EGCG, myricetin and quercetagetin show a high affinity for SARS-CoV-2 RdRp, an RNA polymerase that produces an RNA strand on the matrix [61].

The incidence of the population SARS-CoV-2 virus depends on exposure to its vectors and the condition of the immune system. With the onset of the current pandemic, attention was paid to the need for immune stimulation with vitamins C and D, zinc and cod liver oil [62]. The potential of polyphenols has also been highlighted due to their antioxidant properties and their ability to seal blood vessels. This induces anti-inflammatory mechanisms, especially through the inhibition of proinflammatory cytokine synthesis. Literature data report that polyphenols in green tea, grapes, berries, citrus and curcumin are recommended for coping with coronavirus infections [63,64]. The most commonly mentioned are resveratrol, EGCG, heaflavin-3-O-gallate, oolonghomobisflavan-A and theasinensin-D [62]. 

The issue of coronavirus nowadays is of serious concern. The analysis of scientific databases has revealed many literature reviews describing the effects of polyphenols as antiviral antioxidants that support the immune system [8,57,58,59,60,65,66,67,68]. 

## 3. Mechanism of Antiviral Activity

The purpose of Table 1 is to show the recent research trends in the use of natural sources of polyphenols in suppressing viruses. The information focuses on determining the composition of natural compounds in polyphenols and the mechanism of their synergetic action on virus cells. The mechanism behind the inhibition by polyphenol extracts depends on the type of virus and the origin of a given compound. Herpes simplex virus (HSV-1, HSV-2) inhibition is strongly related to the NS3 protease; as for the influenza virus, it is related to the effect on the ectodomain of viral HA or inhibiting NA activity. However, the mechanism can vary significantly depending on whether we are dealing with a single polyphenol compound or an extract containing several polyphenol groups and other non-polyphenol substances [69,70]. Most of the studies mentioned in Table 1 indicate that antiviral activity occurs by inhibiting the virus replication or limiting the virus replication in the early stages of infection. Some suggest using polyphenols in the preventive form, which allows blocking viral entry and progeny virion release. It is currently impossible to determine how a given polyphenol or a group of compounds will react to various viruses, as there is still little information about their mechanism. Intensive research in this area is required. 

Many studies, e.g., [41,69,71,72,73], deal with a question of the synergistic effect of polyphenolic compounds and non-polyphenols, which individually show no or much lower antiviral properties, but together can inhibit the life cycle of viruses efficiently. One study [74] showed an interesting application of tannic acid as a substance used in the production of HEPA filters, which prevented the influenza virus infection unconventionally. Nevertheless, polyphenols are not only used against human viruses. Many studies describe the effect of various polyphenols on the tobacco mosaic virus, which suggests that polyphenols might be applied in the treatment of viruses affecting plants. Additionally, those compounds show antiviral activity against animal viruses, as demonstrated in [75], which describes the property of proanthocyanidin A2 (PA2) inhibiting the spread of porcine reproductive and respiratory syndrome (PRRS). 

The most common polyphenols are catechin, tannic acid, gallic acid, resveratrol, EGCG and various types of kaempferol and quercetin. Most of the information on the inhibition mechanism of different viruses of the same compound is related to each other. That tendency indicates a similar behavior of these compounds in various types of configurations. However, these mechanisms have not been thoroughly investigated and are mostly the author’s hypotheses derived from his research. References [69,70,71,72] focus on describing the effect and doses of given natural compounds for virus suppression. These papers indicate the need to research the mechanism and composition of the compounds listed in Table 1 for the possibility of practical use because these works do not contain information on the mechanism of inhibition. Many papers do not describe in detail the mechanism of action of individual polyphenols. Instead, refs. [34,62,63,64,73] show a possibility of a synergistic effect of polyphenol and non-polyphenol compounds on virus cells, which does not occur in individual applications or is significantly weaker compared to complex use in the form of, e.g., natural extracts.

Presented papers indicate new ways of research that can expand the application of polyphenols in farming and agriculture. Attention should be paid to the synergistic effect of polyphenols, possibly enhancing their basic antiviral properties and suggesting new applications.

Table 2 provides information on the mechanism of action of individual polyphenols, which are plants’ main components, showing antiviral properties. Based on the information contained in Table 2, it can be concluded that the mechanism of inhibition for individual polyphenols depends on the type of virus and its structure. Papers regarding the use of resveratrol [42,52,99] indicate the different mechanisms behind the inhibition of the viruses. It has been shown that it affects mainly the virus’ replication cycle by interacting with the active sites of caspase or protein synthesis. In the case of curcumin [60,69,100], gallic acid [101,102] and catechin [103,104], examples of an inhibitory effect on the influenza virus are shown. Those publications point out the different methods of inhibition for each compound. For instance, the curcumin action is centered on reducing viral NA activity and blocking HA activity, where catechin tends to merge with functional sites of the virus, which inhibits M2 protein expression and viral synthesis. Gallic acid tends to interact with Arg152 of neuraminidase protein, which affects the replication cycle. Those pieces of information suggest that there is no close correlation between the activity against viruses and highlights the need for further research. Chlorogenic acid [83,105] and EGCG [25,29,106] exhibit mechanisms of action against various types of viruses, which differ significantly from each other; however, for HBV we can display a typical relationship in terms of inhibition of DNA synthesis. This fact presents the possibility of determining the correlation in the prevention of viruses. EGCG mainly suppresses viral growth by inhibiting the replication stage, where chlorogenic acid binds to HepG2.2.15, HepG2.A64. Those facts are the main reason that an unambiguous determination of the inhibition mechanism is difficult and needs further research. The main element that we are able to determine is the stage that a given polyphenol inhibits: stage of infection or virus replication in human cells. However, those pieces of information cannot be used to determine a specific mechanism of suppression for these compounds in the case of other viruses or their behavior in the presence of other polyphenolic or non-polyphenolic compounds. Those dependencies are shown in Table 1, where the compounds listed below occur and the inhibition mechanisms described are significantly different. This indicates the need for detailed studies, which must be carried out on each individual virus in order to determine the mechanism behind the antiviral activity. Research should be conducted by testing a wider variety of viruses for each polyphenol and then carrying out comprehensive studies of synergic reactions between other compounds in, e.g., plant extracts that change the nature of the virus inhibition mechanics or increase the efficiency of this process.

## 4. Clinical Trials

Reviewing the available literature on the antiviral activity of polyphenols, it can be noted that clinical trials evaluating their efficacy are an integral part of the development of new preparations. In most papers, the authors mainly focus on studies conducted on isolated cells from animal [1] or human organisms [2], but there are also many reports confirming the efficacy of polyphenols in human studies [3].

A study on 92 patients with HIV/AIDS was designed to indicate the relationship of the amount of polyphenol intake on the lipid profiles of infected subjects. The participants were given dark chocolate containing 2148 mg of polyphenols and 3 g of mate tea, which contained 107 g of polyphenols. Consumption of the products for 15 days significantly improved the concentration of lipoprotein cholesterol. This is essential for the cardiovascular protection of patients but does not directly indicate the effect of polyphenols on antiviral activity [4]. Dactavira drug administered in tablet form has also been confirmed to treat HCV in clinical trials. The formulation composed of sofosbuvir, daclatisvir and epigallocatechin gallate was tested against standard therapy (sofosbuvir and daclatisvir). The treatment resulted in a significant decrease in viral load compared to the conventionally treated group. Incorporation of epigallocatechin gallate resulted in disruption of viral entry, which substantially prevents relapse [5].

Many experiments conducted on cells have shown that green tea catechins have health-promoting effects within the reproductive organs [6]. A 12-week-long study showed that an ointment containing green tea polyphenols (Polyphenon E) was effective in treating external genital warts. A positive treatment effect was found in both men and women. Only about 8% of patients experienced mild adverse events [7]. Another study showed that as many as 53% of patients treated with Polyphenon E 15% were completely cured of primary and new anal and genital warts (about 60% of women and 45% of men), while adverse effects occurred only at the application site [8]. Approximately 60% complete removal of warts, with 10% recurrence of the disease, was found using 10% and 15% sinecatechin-containing ointment. In studies, up to 50% of patients showed local mild to moderate adverse reactions [9]. The clinical efficacy of green tea extracts (Polyphenon E; poly E and (-)-epigallocatechin-3-gallate) administered as ointments or capsules to patients infected with human papillomavirus was investigated. It was indicated that as many as 69% of those treated showed a response. The collected data indicated the possibility of treating cervical lesions caused by human papillomavirus with green tea extracts [10]. 

Clinical trials also support the effectiveness of polyphenols as adjuvants during antibiotic therapy. The effect of resveratrol in synergy with amoxicillin on improving the treatment of children with pneumonia was investigated. The treatment failure rate was significantly lower on day 3 than patients who received a placebo [11]. Resveratrol was also studied in combination with the carboxymethyl-β-glucan solution or saline solution. Intranasal application to patients with upper respiratory tract infection (rhinovirus) reduced symptoms compared to the control group [12].

Many preclinical studies also suggest that polyphenols (including quercetin) also have immunomodulatory and antimicrobial effects. However, these are mostly studies with a low coefficient of statistical significance [13]. According to one study, consumption of quercetin does not affect immune function, but reduces the incidence of upper respiratory infections after intense exercise [14]. Other information was provided in another paper where a study was conducted on approximately 1002 patients. The study did not confirm significant differences between those taking quercetin and patients treated with a placebo [15]. 

The in vitro evaluation of individual polyphenols is an essential step in studying the effect of compounds on antiviral activity. Although research on selected polyphenols is already advanced, detailed information on the impact of most compounds on their antiviral activity confirmed in clinical trials is still lacking. Current evidence for the efficacy of polyphenol supplementation in treatment is promising but mostly insufficient.

## 5. Methods of Polyphenol Isolation

The isolation of polyphenols in the initial stage is determined by the type (extractable or non-extractable) and their future application. Green isolation methods are applied for plant extracts. Therefore, extraction (of varying complexity and advancement) and hydrolysis of plant material (acid or enzymatic, especially for non-extractable polyphenols) are most commonly implemented. A summary of polyphenol isolation and determination methods is described in Table 3. 

The first step in the isolation of polyphenols is the sample preparation. In addition to grinding the plant to pieces/particles of appropriate size, it is often necessary to remove compounds that could be coextracted, but their presence is not desired. This mainly concerns lipophilic compounds removed by organic solvents such as a mixture of petroleum ether and ethyl acetate [108], dichloromethane [109] or n-hexane [110]. With ethyl acetate, it is also possible to remove extractable polyphenols when isolation of non-extractable polyphenols is expected [111]. 

A review of the literature indicates that classical solvent extraction is most commonly applied. This process is performed with water, organic solvents (ethanol, acetone, methanol, formic acid) or mixtures in different volume ratios. Extraction can be carried out over a wide range of time, from 10 min to 24 h [112,113]. A major limitation of the process with organic solvents (especially toxic ones, such as acetone or methanol) is the necessity to remove the extractant before the extracts can be used for pharmaceutical purposes. Vacuum distillation under reduced pressure is most frequently implemented [114]. This solution ensures effective purification of the extract and the process conditions (p, T) do not cause polyphenol degradation. However, an additional unit process increases production time and cost [115]. A special case of classical extraction is the maceration of plant material, which is carried out at ambient temperature. This process does not require an elevated temperature that could affect the polyphenol isolation efficiency, so it is increased by an appropriately selected solvent [114].

An advanced technique that belongs to the group of green methods is supercritical fluid extraction. On the industrial scale, carbon dioxide, water, ethanol or their mixtures are used. Under appropriate pressure/temperature conditions (higher than critical values) CO_2_ is put into a supercritical state, obtaining liquid and gas properties simultaneously. It is considered that this method is suitable for materials containing thermosensitive polyphenols [116]. The added value of SFE is the high purity of the extracts, which are free of extractants because as the system (p, T) is returned to ambient conditions, the extractants (mainly CO_2_) separate from the extract spontaneously. This method is highly efficient and the residence time of one batch is short. However, special equipment and infrastructure and a large financial investment (adequate pressure is costly) are needed to produce supercritical fluids [117].

Increasing the efficiency of conventional extraction is carried out with the assistance of ultrasound or microwaves. It is assumed that these enhancement methods affect the breakdown of plant cells, which facilitates the leaching of bioactive compounds from the plant matrix. Although these methods provide higher yields and significantly reduce extraction time, they must be well optimized before a large-scale implementation [108,122]. The application of ultrasounds can lead to the formation of reactive oxygen species and free radicals, causing the breakdown of polyphenols. Elevated temperatures also cause this phenomenon. On the other hand, in the technique utilizing microwaves, an increase in temperature is observed during the process, and therefore the power of the microwaves must be experimentally selected in relation to the extracted material and the extractant used [117].

Green extraction methods include pressurized liquid extraction (PLE) and pressurized hot water extraction (PHWE). Increasing pressure and therefore increasing temperature leads to a decrease in the viscosity of the extractant, which affects the polyphenol leaching efficiency [123,124]. These methods do not require complicated instrumentation, thus the cost of the process is low. This cost is reduced, especially in PHWE, where the extractant is water, making this method the most ecologically friendly [117]. The reduction of organic solvents eliminates the need to purify extracts (before medical use) and facilitates separation and analytical procedures [124]. However, it should be noted that the choice of temperature (above the boiling point, but below the critical point) requires optimization with respect to the extracted polyphenols from specific biomass. Too high a temperature can lead to the degradation of polyphenols [117].

Literature data also describe the utilization of acid or enzyme-induced hydrolysis. These methods are applicable to problematic materials, such as olive waste or to isolate non-extractable polyphenols. These cases are dedicated to difficult matrices that contain a large amount of secondary compounds (e.g., fats) or are required by the structure of the material [111,118].

The literature also describes the potential of using a new generation of green solvents, called natural deep eutectic solvents (NADES). These are mixtures of basic plant metabolites, such as sugars, amino acids, alcohols, organic acids, amines or choline derivatives, which exhibit the properties of ionic liquid and deep eutectic solvents [126]. The potential of NADES in polyphenol extraction is due to their unique properties—viscosity, polarity, and formation of hydrogen bonds with polyphenols [127]. 

It is estimated that, compared to classical methods, extraction with NADES results in higher yields. This has been proven in the extrusion of *Carthamus tinctorius L.* with various eutectic solvents, including lactic acid–glucose, proline–malic acid and sucrose–choline chloride [128]. In another study, NADES were used to extract the alga *Chlorella vulgaris* to obtain an extract rich in antioxidants (including the polyphenols gallic acid, caffeic acid, p-coumaric acid and ferulic acid). In addition to higher yields, extraction with NADES was also found to result in increased antioxidant activity of the extracts and selectivity of target antioxidants. Thus, this method allows a more efficient recovery of valuable bioactive compounds from biomass without exposing them to degradation or inactivation [129]. 

The efficiency of polyphenol isolation methods is evaluated by various instrumental analysis techniques via spectrophotometry, mass spectrometry, chromatography or magnetic resonance. In the simplest way, TPC is determined colorimetrically with the Folin–Ciocalteu reagent. This technique uses a color reaction between the polyphenols and the reagent applied, resulting in blue color. The concentration of polyphenols is determined by measuring the absorbance at a wavelength of 750 nm [122]. More advanced systems use chromatography, which both separates the mixture and examines its composition. In the determination of polyphenols, basic thin layer chromatography (TLC) [116], high performance and ultra-performance liquid chromatography (HPLC and UPLC) [113], as well as liquid chromatographs equipped with a mass detector (LC-MS), are used [121]. More advanced methods such as nuclear magnetic resonance NMR or MALDI-TOF-MS are also applicable [111]. For detection of polyphenols, ultraviolet-visible absorption (UV-vis) is widely used. Advanced techniques allow the determination of the total concentration of polyphenols in plant extracts and the identification of individual compounds.

## 6. Conclusions and Future Perspectives

The bioactive compounds in plants and their extracts have antiviral activity and can be used preventively or to fight infections. There are still plant varieties (some exotic) or their edible parts where the polyphenol profiles are not yet identified. Available detection (UV-Vis, NMR, TLC, HPLC, UPLC, LC-MS) and isolation (extraction, hydrolysis) methods are sufficient for these compounds’ quantitative and qualitative determination. 

Recent studies have been centered on examining which components of polyphenolic extracts inhibit viruses of various origins, along with the determination of the inhibitory doses. Preliminary research indicates possible inhibitory mechanisms that are mainly connected to the early stages of infection. The most frequently indicated effects of polyphenols on viruses are the viral replication cycle, reduction in gene expression, changes in the structure of the virus, interaction through RNA-dependent RNA polymerase, etc. Those interactions depend on the virus’ origin, the type of polyphenol compounds used, and their synergistic interactions. An important topic that has been discussed in recent publications is the synergistic interaction of two or more polyphenols, which can improve the inhibiting effect, even if they have little or no antiviral activity separately. The unambiguous identification of the inhibitory mechanism of those compounds is the main issue for which comprehensive studies should be carried out to determine the relationship between given groups of substances and their effect on viruses.

An innovative approach is the synthesis of polyphenols based on the use of components of natural origin and the combination of synthetic and natural components. The use of such treatments increases the bioavailability of the ingredients, thus increasing the preparations’ effectiveness. Currently, in vitro and in vivo studies have been mostly limited to cellular or single organism tests. There is no confirmation of the effectiveness of polyphenols in clinical trials. Such efforts would also allow a better evaluation of their absorption, metabolism and excretion in the human body and exclude side effects, about which little is known.

## Figures and Tables

**Table 1:** Antiviral activity of different polyphenols extracted from plants.

Material	Polyphenols	Virus	Dose	Mechanism	References
mulberry (*Morus alba*) juice	caffeic acid, chlorogenic acid, p-coumaric acid, cyanidin-3-glucoside, cyanidin-3-rutinoside, 3,4-dihydroxybenzoic acid, gallic acid, rutin	IAV, IBV variety (A/Brisbane/59/2007- [H1N1, BR59], A/Korea/01/2009—[H1N1, KR01], A/Brisbane/10/2007—[H3N2, BR10], B/Florida/4/2006 [FL04])	mulberry juice at 2% and 4% exhibited 1.3 log inhibition on FL04 virus in the pretreatment and cotreatment of the virus	antiviral activity at the initial stage of the virus inhibits the attachment of viral surface protein to its cellular receptor or due to internalization of cell surface receptors from virions and prevents virus adsorption to host cells	[11]
black tea	theaflavin (TF1), theaflavin-3′-monogallate(TF2), theaflavin-3-3′-digallate(TF3)	HCV	EC50—17.89 μM (10.09 μg/mL), (TF1), CC50—442.8 μM (250 μg/mL), (TF1), EC50—4.08 μM (2.92 μg/mL), (TF2), CC50—348.8 μM (250 μg/mL), (TF2), EC50—2.02 μM (1.75 μg/mL), (TF3), CC50—287.7 μM (250 μg/mL), (TF3)	polyphenols prevent cell surface attachment or receptor binding by acting on viral particles and thus inhibiting the cell-to-cell spread	[25]
*Euphorbia cooperi* (euphorbiaceae), *Morus alba* (moraceae)	1.catechin-7-gallate, 2. gallic acid, 3. kaempferol 3-O--(6′′-O-galloyl)-glucopyranoside, 4. quercetin3-O--(6′′-O-galloyl)-glucopyranoside, 5. curcumin, 6. quercetin, 7. kaempferol	HSV-1	CC50—[μg/mL]: 1. 43.2 ± 2.3, 2. 49.8 ± 0.4, 3. 124.1 ± 1.2, 4. 175.6 ± 0.9, 5. 49.8 ± 0.4, 6. 78.1 ± 0.8, 7. 76.1 ± 0.2	-the mechanism inhibits viral replication or viral genome synthesis. HSV-1 employs glycosaminoglycan (GAG) as initial attachment receptors during infection of their host cell, so polyphenols target HSV-1 glyco-proteins -this type of interaction prevents the virus from connecting with binding receptors and cell surface	[33]
plant-based polyphenols	tannic acid with modified silver nanoparticles (TA-AgNPs)	HSV-2	5 μg/mouse TA-AgNPs sized 33 nm applied upon the mucosal tissue	-the mechanism is based on two properties of used compounds -one is connected with tannic acid that interferes with the viral adsorption mechanism - second is based on the ability of silver nanoparticles that can block the attachment of the virus and its entry -nanoparticles can also induct antiviral cytokine and chemokine production -efficiency of TA-AgNPs of the inactivation of the virus might depend on the proline content in HSV glycoproteins	[34]
spice and common food colorant, turmeric	curcumin (Cur), monoacetylcurcumin (MAC)	IAV variety (H1N1)	30 μM in MDCK cells for single compound, 15 μM mixture of polyphenols (7.5 μM Cur and 7.5 μM MAC)	-both polyphenols indicate the various mechanisms of inhibition. Cur and MAC reduced viral NA activity, but MAC did not block HA activity, compared to Cur -MAC dampened phosphorylation, which is essential for efficient IAV propagation -replication of IAV in cells is most likely connected with PI3K/AKT activation, which is suppressed by MAC -both polyphenols inhibit IAV separately but together give better results	[68]
cranberry (*Vaccinium macrocarpon aiton*) extracts	A-type proanthocyanidins (A-PAC), dimers and trimers	IAV, IBV	based on the type of fraction: CC50 ≥ 200 μg/mL (1,2,5), CC50—149.7 ± 3.1 μg/mL (3), CC50—136.4 ± 2.4 μg/mL (4), IC50—50 μg/mL (1,2,5), IC50—5.02 ± 1.2 μg/mL (3 IAV), IC50—3.24 ± 1.4 μg/mL (4 IAV), IC50—6.02 ± 1.2 μg/mL (3 IBV), IC50—4.07 ±1.8 μg/mL (4 IBV)	-PAC-A2 achieves the inhibitory effect of polyphenol extract against influenza viruses interacting with the ectodomain of viral HA and the formation of three hydrogen bonds Phe99, Asn210, and Trp234 of HA, which leads to extensive cross-linking of the IV glycoprotein -those properties affect the HA protein band by reducing its intensity	[69]
green tea, cranberry plant	EGCG, the nutraceutical CystiCranÒ-40 (containing 40%), A-PAC, B-PAC)	coliphage T4II (phage T4),the rotavirus strain SA-11(RTV)	mixture of 60 μg/mL (EGCG) and 100 μg/mL(C-40) or 30 μg/mL (EGCG) and 25 μg/mL (C-40)	the combination of those polyphenols is most likely based on blocking or altering viral ligands or antigenic determinants, which reduce the binding ability of the virus	[70]
adlay tea	the present publication concludes two options for antiviral activity of the given extract, either the polyphenols are non-flavonoid phenols and/or the antiviral activity is elicited by other types of compounds	IAV, IBV variety (A/PR/8/34, H1N1, H3N2 and B)	IC50—2.11–5.13 mg/mL (IAV) IC50—2.91–4.61 mg/mL (IBV) CC50 ≥ 40 mg/mL	antiviral activity most likely manifests itself in the inhibition of virus adsorption to the cell and its replication, where tea also affects binding to cells directly	[71]
*Arachis hypogaea (L.)* skin, ethanol extract	extract most probably consist of phenolic acids (coumaric, ferulic, chlorogenic, p-hydrobenzoic acids), phenolics (catechins, A-PACB-PAC), and stilbenes (resveratrol), where resveratrol is the main compound	IAV, IBV	IC50—1.0–1.5 μg/mL, CC50—5.4–9.1l μg/mL	-the mechanism is probably based on inhibition of replication or its early stages, where a combination of an extract with oseltamivir and amantadine prove to be more effective against the influenza virus -this phenomenon is most probably due to targeting different phases of replication	[72]
plant-based extract	tannic acid (TA)	IAV variety (H1N1)	HEPA filter treated with 5 mg/mL of TA for 2 h at 22 oC	linking TA molecules with HA, which are present on the influenza virus’ surface, allows their interaction and subsequent inhibition of viral proteins NA and M2 in the inner part of the virus	[73]
tea polyphenols found in fruits, nuts and seeds	PA2	PRRS	EC50—2.2–3.2 μg/mL, IC50—2.5–3.2 μg/mL (Marc-145 cells), CC50—126.5 (Marc-145 cells) and 63.9 (PAM cells) μg/mL	inhibition consists of blocking viral entry and progeny virion release, which was obtained from reducing gene expressions of cytokines (TNF-α, IFN-α, IL-1β, IL-6)	[74]
*Ajuga iva (L.)* aerial part extracts	total phenolic content (TPC) 28.3 ±1.12, flavonoids content (FC) 10.5 ± 0.83, tannins content (TC) 7.2 ± 0.31	coxsackie virus type B-3 (CV-B3)	IC50—0.43 ± 0.03 mg/mL, AC50—182 ± 12 μg/mL CC50—2810 ± 36 μg/mL	-	[75]
*Juglans regia*, pellicle extract (WPE)	protocatechuic acid, gallic acid, ellagic acid, quercetin, myricetin, chlorogenic acid, kaempferolarabinoside, avicularin, (+)-procyanidin B2, rutin	HSV-1, HSV-2	ID50—10 µg/mL (HSV-1) ID50—8 µg/mL (HSV-2)	-	[76]
*Cornus canadensis*	1,6-di-O-galloyl-β-D-glucopyranose, 1,2,3-tri-O-galloyl-β-D-glucopyranose, 1,2,6-tri-O-galloyl-β-D-glucopyranose, 2,3,6-tetra-O-galloyl-β-D-glucopyranos, 2,3,4,6-penta-O-galloyl-β- D-glucopyranose, tellimagrandin I, tellimagrandin II, ethyl gallate, caffeic acid, astragalin, isoquercetin, trifolin, kaempferol 3-O-β-D-xylopyranoside, reinutrin, juglanin, avicularin, juglalin, benzyl 2-O-β-glucopyranosyl-2,6-hydroxybenzoate, byzantionoside B	HSV-1	direct mode EC50—11–17 μg/mL (extract) EC50—2.6 ± 0.1 μM (tellimagrandin I) EC50—7 ± 4 μM (2,3,6-tetra-O-gal-loyl-β-D-glucopyranose) EC50—10 ± 2 μM (2,3,4,6-penta-O-galloyl-β-D-glucopyranose) EC50—7 ± 1 μM (tellimagrandin II) absorption mode EC50—9 μg/mL (extract) EC50—5.0 ± 0.2μM (tellimagrandin I) EC50—11 ± 3 μM (2,3,6-tetra-O-gal-loyl-β-D-glucopyranose) EC50—12 ± 4 μM (2,3,4,6-penta-O-galloyl-β-D-glucopyranose) EC50—11 ± 3 μM (tellimagrandin II)	-	[77]
magnolia officinalis	honokiol, magnolol	murine norovirus (MNV-1), feline Calicivirus	5 mg/mL added to food products such as milk or plum, orange, apple juice	-	[78]
pomegranate peel extract	ellagic acid, punicalin, gallic acid, punicalagin	SARS-CoV-2	-	-polyphenols combined with the amino acids contained in S-glycoprotein, ACE2, furin, TMPRSS2 using mainly hydrogen bonds -in punicalagin and punicalin, there are 3-4 hydrogen bonds, and also Pi-alkyl, Pi-Pi bonds are present, which stabilizes the complex	[79]
blueberry	B-PAC	aichi virus	5 mg/mL	various mechanisms are considered in the treatment of host cells with B-PAC, including binding: -host cells receptors -virus particles and blocking attachment -host cell and preventing host cell entry connected to blocking/damaging the above host cell or viral receptor, which prevents viral entry	[80]
extracted plants	type of the flavonoids	dengue virus (DENV)	-	-inhibition of this virus is caused by the disruption of the NS2B-NS3 protein complex, which has a negative effect on replication -each of the present flavonoids interacts with different amino acids of viral protein, which changes the process mechanism:	[81]
*Euphorbia lunulate*	quercetin 3-O-(2′′,3′′-digalloyl)-β-D-galactopyranoside			Gly 87, Val 146, Asn 167, within the active site	
*Sedum sarmentosum*	quercetin 3-O-α-(6′′-caffeoylglucosyl-β-1,2-rhamnoside)			Lys 74, Ile 165 within the inactive site	
*Pas* *siflora tripartite*	schaftoside			an arene–arene link with amino acid Trp 83	
*Myrica rubra*	myricetin			Trp 83 Gly 87 and Val 146	
*Anethum graveolens*	quercetin 3-sulfate			an arene cationic link with Lys 74	
*Citrus lumia, Cyclopia, Subternata*	eriocitrin			Lys 74	
*Richilia catigua*	catiguanin B			a cationic arene interaction with Lys 74 and a hydrogen bond donation with Trp 83	
*Lepisorus contortus*	4′,5,7-trihydroxy-3-methoxyflavone-7-O-α-L-arabinofuranosyl(1→6)-β-D-glucopyranoside			Asn 167, Val 147, Trp 89	
*Scutellaria baicalensis*	wogonin 7-O-β-D-glucuronide			Gly 87, Trp 83	
*Silybum marianum*	silychristin			a cationic arene link with Lys 74 and a hydrogen bond with Trp 83	
plant-based polyphenols	catechin, procyanidin B2 (PB2)	felinecalici virus (FCV F9), MNV-1	0.5 and 1 mg/mL for both compounds, where time was the main factor deciding about inhibition of the viruses	-inhibition is achieved by PB2 that changes the structure of the P domain of VLPs -PB2 binds the P domain, where electrostatic interactions play a dominant role while PB2 significantly alters tertiary but not secondary structures of VLPs	[82]
grapefruit mesocarp extract	hesperidin	HCV genotype 3a	IC50—23.32 µmol/L	the mechanism is based on HCV NS3 protease fused with co-factor NS4A, which then binds hesperidin with the catalytic site residues of the NS4A-NS3 protease domain	[83]
almond skin extracts	EC, eriodictyol quercetin, catechin protocatechuic acid, kaempferol p-hydroxybenzoic acid chlorogenic acid vanillic acid isorhamnetin naringenin trans-p-coumaric acid eryodictiol-7-O-glucoside isorhamnetin-3-O-glucoside isorhamnetin-3-O-rutinoside naringenin-7-O-glucoside kaempferol-3-O-glucoside kaempferol-3-O-rutinoside quercetin-3-O-glucoside quercetin-3-O-galactoside quercetin-3-O-rutinoside	HSV-1	0.4 mg/mL extracts concentration (90% decrease of viral titer)	NS acts on the HSV-1 lytic cycle in that it blocks virion entry into the cells, i.e., the polyphenols bind to cell receptors of the virus, preventing them from entering the cell	[84]
141 diverse plant and fungal species belonging to 66 different families, with asteraceae (10%), lamiaceae (10%), apiaceae (6%), and fabaceae (4%)	1. rutamari, 2. piperine, 3. piperylin, 4. 1-[(2E,4E,8E)-9-(1,3-benzodioxol-5-yl)-1-oxo-2,4,8-nona-trienyl]-pyrrolidine, 5. piperoleine A, 6. dehydropipernonaline, 7. pipernonaline, 8. chabamide, 9. ganoderol B	Hong Kong/68 (HK/68), rhinovirus RV-A2, CV-B3	extracts: IC50—50 μg/mL (HK/68) IC50—20 μg/mL (CV-B3) IC50—11 μg/mL (RV-A2) pure compound: 1. CC50—4.7 μM (MDCK cells) and 4.6 μM (HeLa cells), 2. CC50—50 μM (HeLa cells), 3. CC50 ≥ 100 μM (HeLa cells), 4. CC50 ≥ 100 μM (HeLa cells), 5. CC50—25 μM (HeLa cells), 6. CC50—34 μM (HeLa cells), 7. CC50—21 μM (HeLa cells), 8. CC50—11 μM (HeLa cells), 9. CC50 ≥ 100 μM (MDCK cells) and >100 μM (HeLa cells), 1. IC50—2.7 μM (HK/68 in MDCK cells) and 5.1 μM (CV-B3 in HeLa cells), 2. IC50—41 μM (RV-A2 in HeLa cells), 3. IC50—51 μM (CV-B3 in HeLa cells) and 79 μM (RV-A2 in HeLa cells), 4. IC50—61 μM (CV-B3 in HeLa cells), 5. IC50—22 μM (CV-B3 in HeLa cells), 6. IC50—24 μM (CV-B3 in HeLa cells), 7. IC50—32 μM (CV-B3 in HeLa cells), 8. IC50—9.1 μM (CV-B3 in HeLa cells), 9. IC50—17 μM (HK/68 in MDCK cells) and 65—μM (RV-A2 in HeLa cells),	the paper indicates the properties of ganoderol B to inhibit RV coat protein, which is connected to its antiviral activity	[85]
*Canarium album*	isocorilagin	IAV variety (H1N1)	IC50—9.19 ± 1.99 µM A/Puerto Rico/8/34 (H1N1) IC50—23.72 ± 2.51 µM A/Aichi/2/68 (H3N2) IC50—4.64 ± 3.01 µM for NA-H274Y (H1N1)	isocorilagin inhibiting NA activity in a dose-dependent manner via residues Arg118, Glu119, Arg156, and Glu276. Interaction with NA mainly through hydrogen bonds and van der Waals forces	[86]
rhoeo discolor leaves and their methanol extract	luteolin-7-glucoside 15% kaempferol 75% isoquercetin 5% quercetin 2% rutin 3%	IAV variety (H1N1)	CC50—0.90 ± 0.01 μg/mL IC50—0.30 ± 0.02 μg/mL	-the inhibition of the influenza virus is accomplished through the suppression of the HA - MF1 fraction acts at the HA level and thus prevents the virus from binding to the cell surface receptors	[87]
flos caryophylli	rhamnetin-3-O-b-D-glucuronide-600-methyl ester, rhamnazin-3-O-b-D-glucuronide- 600-methyl ester, kaempferol-3-O-b-D-glucuronide-600-methyl ester, isorhamnetin-3-O-b-D-glucuronide-600-methyl ester, rhamnetin-3-O-b-D-glucopyranoside, quercetin 3-O-b-D-glucuronide-600-methyl ester, quercetin 3-O-b-D-glucuronide, isoquercitrin, isorhamnetin-3-O-b-D-glucopyranoside, rhamnazin-3-O-b-D-glucopyranoside, 1,2,3-tri-O-galloylglucose, casuarinin, tellimagrandins I, 1,3-Di-O-galloyl-4,6-HHDP-glucose, casuarictin, eugeniin, 1,2,3,6-tetra-O-galloylglucose, isobiflori, biflori	IAV variety (H1N1)	IC50—8.4 to 94.1 μM EC50—1.5–84.7 μM CC50—374.3–1266.9 μM	inhibition of the key surface proteins of the virus, which is NA, is affected by a group of polyphenols	[88]
*Vaccinium oldhamii* ethanol extracts	procyanidin B2,O-hexosides, quercetin-3-O-rhamnosidequercetin-O-pentoside-O rhamnoside	IAV, IBV	IC50—38 µg/mL (30% extract) IC50—22 µg/mL (40% extract) IC50—65 µg/mL (50% extract) CC50—251 µg/mL (30% extract) CC50—160 µg/mL (40% extract) CC50—78 µg/mL (50% extract)	ferulic acid and its derivatives bind NA and inhibit the initial stage of IFV infection, where quercetin and rhamnoside suppress IFV replication in cells	[89]
*Solieria filiformis*, *Ecklonia arborea*	solieria filiformis: kaempferide, kaempferol-3-O-rutinoside, quercetin 3-O-malonylglucoside, demethoxycentaureidin7-O-rutinoside, quercetin 3-(6-O-acetyl-beta-glucoside) ecklonia arborea: phlorofucofuroeckol-B, formononetin, apigenin 7-O-glucoside	measles virus	solieria filiformis: CC50 ≥ 1500 μg/mL IC50 -0.4 ± 0.11 μg/mL ecklonia arborea: CC50 ≥ 1500 μg/mL IC50—2.6 ± 0.28 μg/mL	inhibition is based on the deactivation of the virus virion, which prevents it from absorbing and penetrating the host cell	[90]
blueberry	B-PAC	MNV-1, FCV-F9	5 mg/mL in simulated intestinal fluid	-in used nutritional models, milk has decreased antiviral activity because of the presence of complex matrices containing lipids -the publication clarifies that the presence of proteolytic enzymes did not affect the inhibition of those viruses	[91]
plant based polyphenols	EGCG, TF1, theaflavin -3′-O-gallate (TF2a), theaflavin-3′-gallate (TF2b), TF3, hesperidin, quercetagetin, myricetin	SARS-CoV-2	maximum tolerated dose for humanEGCG—0.441 (log mg/kg/day) TF3—0.438 (log mg/kg/day) TF2b—0.438 (log mg/kg/day) TF2a—0.439 (log mg/kg/day)	the complex is formed by van der Waals bonds, electrostatic interactions, nonpolar solvation free energy, where polyphenols bind to the virus through RNA-dependent RNA polymerase (RdRp)	[92]
*Cassia alata*	alatains A and B	tobacco mosaic virus (TMV)	IC50—18.8 µM (A type) IC50—11.4 µM (B type)	the presence of C-14—C-5 linkage between a chromone moiety and an isocoumarin moiety in the studied polyphenols	[93]
eucalyptus bark extract	benzoic acid, quinol, salicylic acid, myricetin, rutin	TMV	100 μg/mL	the direct inhibition of virus replication as well as by simultaneous activation of the host innate immune response and inducing SAR against the virus	[94]
plant-based polyphenol	resveratrol	vaccinia virus (VACV), monkeypox virus (MPXV)	CC50—176.88 ± 17.44 μM (VACV HFF cells),IC50 -3.51 ± 1.22 μM (VACV HFF cells), CC50—157.75 ± 23.66 μM (VACV Hela cells), IC50—4.72 ± 2.34 μM (VACV Hela cells), IC50—12.41 μM (MPXV-WA), IC50—15.23 μM (MPXV-ROC)	polyphenol affects genome replication as well as post-replicative gene expression, where resveratrol affects the cellular function of VACV	[95]
plants and natural products based on polyphenols	delphinidin (D), EGCG	west Nile virus (WNV), zika virus (ZIKV), DENV	antiviral activity is dose-dependent with values ranging from 1–10 μM, the best results were obtained with 10 μM	inhibition mechanism is based around entry steps of the virus life cycle, where polyphenols contain trihydroxyphenyl group at R2, which may interact with the function of proteins at multiple binding sites	[96]
fruits and vegetables (plant-based polyphenols)	isoquercitrin (quercetin-3-O-glucoside or Q3G)	ZIKV	IC50—15.5 ± 2.3 μM (A549), CC50—551.2 ± 43.2 μM (A549), IC50—14.0 ± 3.8 μM (Huh-7), CC50—326.8 ± 45.7 μM (Huh-7), IC50—9.7 ± 1.2 μM (SH-SY5Y), CC50—582.2 ± 41.4 μM (SH-SY5Y),	polyphenol prevents the internalization of virus particles into the host cell, most likely by clathrin-mediated endocytosis pathway involving Axl/Gas6 as entry factors	[97]
honey, tea, red wine	pinocembrin	ZIKV	IC50—17.4 µM	polyphenol inhibits the replication cycle of the virus as well as RNA production and envelope protein synthesis, where those actions happen in the post-entry stages of the infection	[98]

CC-cytotoxic concentration, EC—effective concentration, ID50-infective dose.

**Table 2:** Antiviral activity mechanism of individual polyphenols mostly found in plants.

Polyphenol	Virus Type	Activity Mechanism	Refs.
quercetin	SARS-CoV-2	interaction with Spike occurs between amino acid Thr 445, Ile 446; as for main protease it binds to Thr 26 superior main protease docking result compared to spike docking, better inhibitory effect on replication cycle of the virus rather than penetration/adsorption cycle	[16]
resveratrol	Epstein–Barr virus	decreasing levels of reactive oxygen species, blocking protein synthesis and inhibiting virus-induced activation of transcription factors, which affects replication of the individual virus	[42]
	rotavirus	inhibition of the replication in the Caco-2 cell line	[52]
	vesicular stomatitis virus	suppression of the spread of the virus by interaction with the active sites of caspase-3 and -7	[99]
curcumin	SARS-CoV-2	entry into host cells is also blocked by blocking the enzyme ACE2; curcumin has a high affinity for ACE2 ligands	[60]
	influenza virus	reduction of viral NA activity and blocking HA activity	[69]
	SARS-CoV-2	inhibition due to interaction with Mpro receptor of SARS-CoV-2, which occurs by binding with amino acid Thr26, His41, Gln189	[100]
EGCG	HCV	suppressing by blocking virus entry via viral envelope proteins and inhibiting cell-to-cell transmission	[25]
	HBV	inhibition of DNA synthesis during virus replication	[29]
	the duck Tembusu virus (DTMUV)	reduction of the viral infection in BHK-21 cells, expressions of the viral E protein and virus titers. EGCG affects the adsorption step of the infection and replication stage of the virus in BHK-21 cells	[106]
chlorogenic acid	infectious bursal disease virus	inhibiting histamine production, NF-kB activation, which affects the production of the pro-inflammatory cytokines TNF-a and IL-1b	[105]
	HBV	inhibiting DNA of the virus by binding to HepG2.2.15 and HepG2.A64	[107]
catechin	influenza A virus	binding to functional sites PHE47A and LEU43A, which inhibits M2 viral mRNA synthesis as well as M2 protein expression	[103]
	dengue virus	interaction with NS5 protein, by binding to amino acids Asn609, Asp663, His798	[104]
gallic acid	influenza A virus	inhibition of replication of the virus, by binding to Arg152 of neuraminidase protein	[101]
	paramyxoviruses	affects replication cycle of the virus by inhibiting ribonucleotide reductase enzyme	[102]

**Table 3:** Methods of polyphenol isolation and determination.

Material	Pretreatment	Polyphenol Isolation	Time	Polyphenol Determination	References
soursop leaves	-	water and ethanol/water (70:30 *v*/*v*) extraction	10–20 min	HPLC	[112]
olive waste	-	ultrasound-assisted enzyme catalyzed hydrolysis	-	1H NMR and 13C NMR	[118]
*Heliotropium taltalense*		methanol extraction in an ultrasonic bath	1 h	UPLC	[119]
maritime pine	removing lipophilic compunds with a petroleum ether/ethyl acetate (50:50 *v*/*v*) mixture	ethanol/water (85:15 *v*/*v*) extraction	2 h	LC-MS and NMR	[108]
*Cuspidaria convoluta*	-	methanol maceration	24 h	UV-VIS and HPLC-MS/MS	[114]
*Gaultheria phillyreifolia* and *G. poeppigii* berries	-	methanol/formic acid (99:1 *v*/*v*) extraction	-	HPLC	[120]
green tea	-	ethanol/water (70:10, *v*/*v*) extraction in ultrasonic cleaner	1 h	HPLC and LC-MS	[121]
*Aronia melanocarpa*	defatting with n-hexane and with dichloromethane	methanol/acetic acid (19:1, *v*/*v*) extraction with stirring	8 h	HPLC	[110]
Saharan myrtle tea	-	methanol/water (80:20, *v*/*v*) extraction	3 ∙ 24 h	UPLC	[113]
*Syzygium alternifolium*	removing lipophilic compunds with a dichloromethane	methanol/water (80:20, *v*/*v*) or acetone/water (80:20 *v*/*v*) extraction with sonification	15 min	UV-VIS	[109]
pomegranate peels	removing of extractable polyphenols using ethyl acetate	non-extractable polyphenols obtained via acid hydrolysis (6M HCl)	2 h	TLC, CC, NMR, MALDI-TOF-MS	[111]
grape processing lees	-	supercritical fluid extraction (SFE) with 90% of supercritical carbon dioxide and 10% (*w*/*w*) of ethanol	10 min	TLC and HPLC	[116]
*Myrtus communis L.* leaves	-	extraction with aqueous ethanol with assistance of microwaves	30–90 s	Folin–Ciocalteu colorimetric method	[122]
goldenberry	-	ethanol/water solution (70:30, *v*/*v*) pressurized liquid extraction (PLE)	10–60 min	HPLC-DAD	[123]
grape pomace	-	pressurized hot water extraction (PHWE)	5 or 30 min	MALDI-TOF-MS	[124]
*Phyllanthus Emblica*	-	soxlet extraction with ethanol/water (7:3, *v*/*v*)	30 min	Folin–Ciocalteu colorimetric method	[125]

TLC—Thin Layer Chromatography, UPLC—Ultra Performance Liquid Chromatography, UV-VIS—Ultraviolet–Visible Absorption, LC-MS—Liquid Chromatography—Mass Spectrometry, HPLC—High Performance Liquid Chromatography NMR—Nuclear Magnetic Resonance, MALDI-TOF-MS—Matrix-Assisted Laser Desorption/Ionization—Time-Of-Flight Mass Spectrometry, HPLC-DAD—High-Performance Liquid Chromatography—Diode-Array Detection.

## Abbreviations

B-PAC	B-type proanthocyanidins
CV-B3	coxsackie virus type B-3
CC	cytotoxic concentration
DENV	dengue virus
DTMUV	duck Tembusu virus
EC	effective concentration
ECG	epicatechin gallate
EGCG	epigallocatechin-3-gallate
FCV	felinecalici virus
HA	hemagglutinins
HAV	hepatitis A virus
HBV	hepatitis B virus
HCV	hepatitis C virus
HPLC	high performance liquid chromatography
HPLC-DAD	high performance liquid chromatography—diode-array detection
HSV-1	herpes simplex virus 1
HSV-2	herpes simplex virus 2
IAV	influenza virus A type
IC	inhibitory concentration
IBV	influenza virus B type
ID	infective dose
LC-MS	liquid chromatographs equipped with a mass detector
MALDI-TOF-MS	matrix-assisted laser desorption/ionization—time-of-flight mass spectrometry
MNV-1	murine norovirus
MPXV	monkeypox virus
NA	neuraminidase
NADES	natural deep eutectic solvents
NMR	Nuclear Magnetic Resonance
PA2	proanthocyanidin A2
PAC-A	A-type proanthocyanidins
PHWE	pressurized hot water extraction
PLE	pressurized liquid extraction
PRRS	porcine reproductive and respiratory syndrome
RV	rhinovirus
SARS	severe acute respiratory syndrome
TF1	theaflavin
TF2	theaflavin-3′-monogallate
TF2a	theaflavin-3′-O-gallate
TF2b	theaflavin-3′-gallate
TF3	theaflavin-3-3′-digallate
TLC	thin layer chromatograph
TMV	tobacco mosaic virus
TPC	total phenolic content
UPLC	ultra performance liquid chromatography
UV-VIS	Ultraviolet–visible absorption
VACV	vaccinia virus
ZIKV	zika virus
